# In vivo degradation and neovascularization of silk fibroin implants monitored by multiple modes ultrasound for surgical applications

**DOI:** 10.1186/s12938-018-0478-4

**Published:** 2018-06-20

**Authors:** Shouqiang Li, Dandan Yu, Huan Ji, Baocun Zhao, Lili Ji, Xiaoping Leng

**Affiliations:** 1 Department of Ultrasound, The Second Affiliated Hospital of Harbin Medical University, 246 Xuefu Road, Nangang District, Harbin, Heilongjiang Province China; 2 The Key Laboratories of Myocardial Ischemia, Harbin Medical University, Ministry of Education, Harbin, Heilongjiang Province China

**Keywords:** Surgical applications, Silk fibroin, Ultrasound, Biodegradation, Neovascularization

## Abstract

**Background:**

In this paper we aimed to investigate the neovascularization and biodegradation of the silk fibroin in vivo using multiple modes ultrasound, including two-dimensional, three-dimensional and contrast-enhanced ultrasound by quantifying the echo intensity, volume and contrast enhancement of the silk fibroin implants.

**Method:**

A total of 56 male Wistar rats were randomly divided into two groups and 4%(w/v) silk hydrogels were injected subcutaneously at hind limb or upper back of the rats respectively to compare the biodegradation rate in different sites of the body. The implants were observed at day 0, 4, 8, 12, 16, 18, 20 with multiple modes ultrasound.

**Results:**

The echo intensity of silk fibroin implants increased and the volume decreased gradually, and complete degradation was confirmed 18 and 20 days after subcutaneous implantation at the upper back and at the hind limb respectively. This demonstrated that the silk fibroin embedded in the upper back degraded slightly faster than that in the hind limb. Additionally, the neovascularization revealed by the contrast enhancement values of CEUS showed that there was a relatively low enhancement (< 5 dB) during day 4 to day 16, followed by moderate enhancement at day 18 (5–20 dB), and a significant enhancement at day 20 (> 40 dB).

**Conclusion:**

This study suggests that multiple modes ultrasound imaging could be an ideal method to evaluate the degradation and neovascularization of biomaterial implants in vivo for surgical applications.

## Background

Silk has been used in biomedical applications for centuries, particularly as sutures. Silk fibroin can be processed into different constructs (e.g. porous scaffolds, films, hydrogels, and nano/microspheres) and therefore can be considered as a useful biomaterial for variety of biomedical applications ranging from tissue repair to drug delivery [1–3]. The ability to make biomaterials suitable for diverse applications is paramount to the success of regenerative medicine. An optimal biomaterial should be tailored for a specific purpose and several available reaction parameters, including the degree of inflammation, the level of vascularization and the degradation rate [4, 5]. The proteolytic degradation of silk hydrogel has a temporal window, which provides options for biomedical applications that require slow material degradation to facilitate tissue regeneration and remodeling. Therefore, monitoring of the silk fibroin during degradation is essential to evaluate its potential clinical application, and the proteolytic degradation in vivo has to be documented. Similar to other biomaterials, the in vivo degradation of silk implants has been assessed mainly by excising the explants followed by histology. Traditional two-dimensional ultrasound (2D US), an ultrasound-based diagnostic imaging technique to visualize internal body structures, has been reported to investigate biomaterial degradation [6–9]. Contrast-enhanced ultrasound (CEUS) is one of noninvasive ultrasound technique applied to clinical practice that can image blood vessels with microbubble-based contrast agents being introduced to the blood stream and thereby enhancing echogenicity [4, 10–13]. Based on the contrast enhancement that is caused by the microbubbles injected in the blood stream, neovascularization in the biomaterial implant during degradation could be assessed. However, there have been few studies reported on using CEUS in nondestructive biomaterial assessment so far. And our previous studies have shown that conventional 2D and CEUS could monitor the degradation and vascularization of silk implants [14]. However, 2D US can only provide the diameter changes of the implants rather than the volume changes. With the latest developments in the field of three-dimensional ultrasound (3D US), it offers advantages over 2D US within a scanned volume [15–17]. Multi-plane, including sagittal, transverse and coronal view scan be depicted and volumes can be measured accurately. Combining 2D and 3D US with CEUS could provide more detailed information on material degradation and tissue regeneration, making ultrasound a more useful technique for biomaterial-based tissue engineering.

## Methods

### Silk purification

Partially degummed silk fibers were purchased from Xiehe Silk Corporation (Suzhou, Jiangsu province, China). The silk fibers were further degummed in the lab to remove residual sericin contaminants as previously described [18]. Briefly, silk fibers were boiled in 0.02 M sodium carbonate solution for 30 min, then drained and dried in a bio-safety cabinet overnight after being rinsed with ultrapure water for three times. The dried fibers were dissolved in 9.3 M lithium bromide solution at 60 °C to obtain a concentration of 20%. To remove the lithium bromide and insoluble fibrous debris, the solution was dialyzed against pure water for 2 days, then the final concentration of purified silk was 4%(w/v). The solution obtained was autoclaved and stored at 4 °C for use. The solution was diluted with water to obtain low concentration silk hydrogels or freeze-dried by a lyophilizer (CHRIST Alpha 2–4 LSC plus, Martin Christ Gefriertrocknungsanlagen GmbH, Germany) and reconstituted with water to obtain high concentrations according to the procedure in the literature [19].

### Polyethylene glycol-silk hydrogel preparation

Defined weight percentage Polyethylene glycol (PEG) solution was prepared by mixing liquid state PEG400 with ultrapure water and stored at room temperature after filtration through a 0.22 µm filter. PEG was gently mixed with silk in a glass vial at a volume ratio of 1:1 to obtain PEG-silk hydrogels. The solution was subjected into syringes for injection. Silk solution was autoclaved prior to the mixing. Autoclaving did not significantly change the gelation time and gel properties, which is similar to our previous report [20].

### Murine experimental design

This study was performed according to the Guide to the Care and Use of Experimental Animals from institutional Animal Research Committee at the Harbin Medical University. A total of 56 male Wistar rats weighing 150–200 g were obtained from the Animal Research Center, the Second Affiliated Hospital of Harbin Medical University. They were fed with normal diet, water was available ad libitum and there was an artificial light–dark cycle of 12 h each. The rats were anesthetized with intraperitoneal 10% chloral hydrate (3 ml/kg) before animal surgery and ultrasound imaging. 28 rats received a silk hydrogel implant subcutaneously at the hind limb and the other group of 28 rats received the implant subcutaneously at the upper back. An approximately 0.2 ml silk hydrogel was injected subcutaneously with a 1 ml syringe. After implantation, 4 rats from each group were selected randomly and underwent US imaging on day 0, 4, 8, 12, 16, 18 and 20 (n = 4). All experiments were performed under aseptic conditions and all rats survived without any complications.

### Two-dimensional US

After surgery, 4 rats were randomly selected from each group and underwent ultrasound imaging every four or 2 days (on day 0, 4, 8, 12, 16, 18, and 20). Traditional 2D US was applied through a L74M transducer of HI VISION Preirus (Hitachi Medical) to distinguish the silk hydrogel implant from the adjacent tissue and to observe the echo intensity as well as the shape of the silk implants. Scanning depth (2 cm), frequency (12 MHz), dynamic range (44 Hz), gain settings (56), and frame rate (19–44 fps) were initially optimized and maintained constant throughout the imaging. The images of silk hydrogel at different time point were acquired for offline analysis. To quantify a textural change of the silk implant, the echogenicity acquired from 2D US images were measured by the image processing software ImageJ (National Institutes of Health, USA). The region of interest (ROI) was delineated and the values were averaged and graded in gray scale using arbitrary units that range from 0 to 255 from black to white.

### Three-dimensional US

Three-dimensional ultrasound images of the silk implant were obtained using a GE E8 machine (GE Medical Systems) with a 3D mode scanner (RSP6-16-D) after the 2D images were acquired. After visualizing the implants in 2D, the 3D mode was activated, and the guiding volume box was placed over the entire silk fibroin implants, then the scanning was completed automatically. The ultrasonic probe was fixed with a fixed frame, so that the position of probe was kept steady and the condition of the instrument were consistent to obtain qualify 3D volume images. The acquired volume images were stored for offline analysis by 3D View software which is integrated into the GE E8 ultrasound system to measure the implant’s volume. The delineations of the regions of interest indicated the silk hydrogel, the 3D values of which volume were assessed by three observers and repeated twice, and the values were obtained as average. All observers were experienced at contour definition with this software.

### Contrast-enhanced ultrasound

Contrast-enhanced ultrasound imaging was performed at 4, 12, 16, 18 and 20 day after surgery with the L12-3 transducer of Philips CX50 Ultrasound Machine (Philips Healthcare). After 3D ultrasound imaging was performed, the Contrast Mode was switch and contrast harmonic mode was applied with a frequency of 8 MHz and a low mechanical index (MI) of 0.1 with frame rate 43 frames/s. CEUS was performed through intravenous administration of SonoVue™ (Bracco, Italy) contrast agent. A vial of Sonovue was diluted with 5 ml saline, yielding the final concentration of 1 × 10^8^ microbubbles/ml. After locating the silk hydrogel implants beneath the rat hind limb skin, 0.2 ml (2.0 × 10^7^) diluted microbubbles were injected into rat’s tail vein as a bolus, followed by a 1 ml saline flush. The ultrasound images were recorded immediately after the injection for 2 min and stored digitally. In order to obtain the best contrast imaging, the probe was kept steady. To quantify the blood supply inside the implants, the peak video intensity (VI), which represents the ultrasound enhancement by microbubbles, was measured with customized MCE software (Jiri Sklenar, University of Virginia, USA).

### Tissue preparation and histology

To evaluate biodegradation of the gel samples and the reaction around the gel samples, the rats were euthanatized by overdose of chloral hydrate after CEUS imaging at corresponding time point. Immediately after death, the biomaterials were explanted together with the adjacent implantation tissue and the silk gel implants were sectioned into two slices. One slice was fixed in 4% buffered formalin for hematoxylin and eosin (H&E) staining and immunohistochemical staining. The other slice was fixed in 2.5% glutaraldehyde-phosphate fixative for electron microscopy.

#### Hematoxylin and eosin (H&E) staining and immunohistochemical staining

The samples were fixed with 10% formaldehyde for 24 h and dehydrated in a series of alcohols, transferred to xylene and embedded in paraffin. A 4 μm thick paraffin section was cut and fixed on a glass slide. After being deparaffinized and rehydrated, the sections were stained with hematoxylin and eosin (H&E) to observe the silk fibroin implants and adjacent tissues. For immunohistochemical microscopy, the sections were treated with 3% hydrogen peroxide in order to block the endogenous peroxidase. To reduce the background staining and nonspecific binding, the sections were blocked with 10% normal horse serum for 1 h. Samples were incubated for 1 h with primary antibodies against endothelial cell surface marker VIII (Boster Biological Technology, 1:250). The sections were observed under optical microscope (BX41, Olympus, Japan) with digital camera unit (DP25, Olympus, Japan) focused on the inside of the implants and the adjacent soft tissues.

#### Transmission electron microscope (TEM)

Transmission electron microscope experiment was performed at Electron Microscope Center of Harbin Medical University. The silk gel implants were fixed by immersing in 2.5% glutaraldehyde-phosphate fixative, rinsed in phosphate buffered saline (PBS), post fixed with 1% osmium tetroxide, and then dehydrated in a graded series of ethanol, and embedded in Epon 812 resin. Semi-thin sections, stained with toluidine blue, were examined by a light microscope for localization. The ultrathin sections were cut and stained with 4% uranyl acetate and 0.5% lead citrate. Ultrastructural observations were made with a Hitachi H7650 transmission electron microscope (Hitachi, Japan) operated at 80 kV.

### Statistics

Data are reported as mean ± standard deviation. Statistical analyses were performed by the SPSS17.0 software. Differences were considered significant at *p *< 0.05. The interobserver reliability for volume assessment by different observers were assessed by intraclass correlation coefficients (ICC). Volume and acoustic intensity at different time points measured by 3D US and contrast ultrasound respectively were compared through one-way ANOVA. Dunnett’s Test was performed to compare results of each time point to determine whether there were significant differences.

## Results

### Degradation of silk gel implants determined by two-dimensional US

**Fig. 1:**
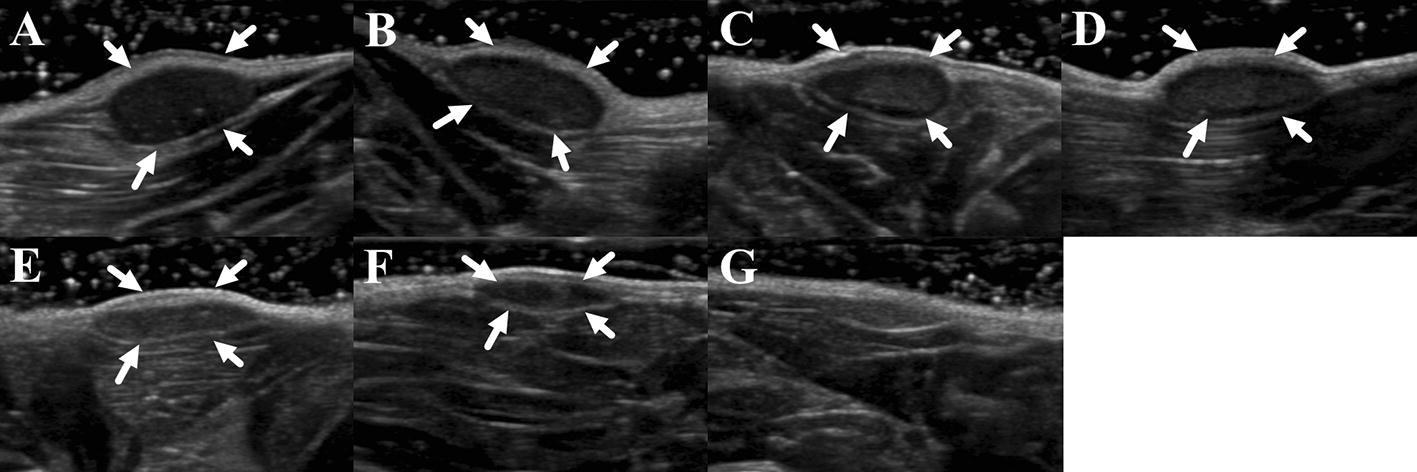
Silk fibroin implants at the hind limb subcutaneous observed by 2D US at day 0, 4, 8, 12, 16, 18, and 20. The implants appeared low-level gray in the first few days and oval shape (**a**–**d**), then the shape of the implants were thinner and flatter from day 16 (**e**–**f**), and the silk hydrogel implants cannot be clearly recognized and the echogenicity equal to the surrounding’s at day 18 (**g**); arrows, outline of silk hydrogel implants

**Fig. 2:**
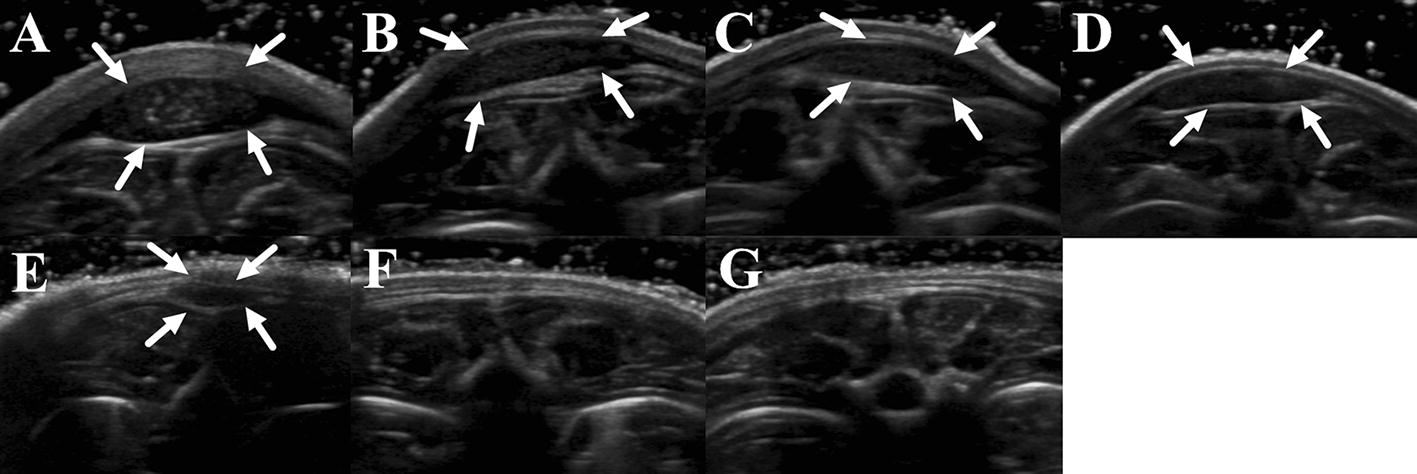
Silk fibroin implants at the upper back subcutaneous observed by 2D US at day 0, 4, 8, 12, 16, 18, and 20 (**a**–**g**). The implants became thinner and flatter from day 4 (**b**–**e**), and cannot be recognized at day 18 and 20 (**f**–**g**); arrows, outline of silk hydrogel implants

**Fig. 3:**
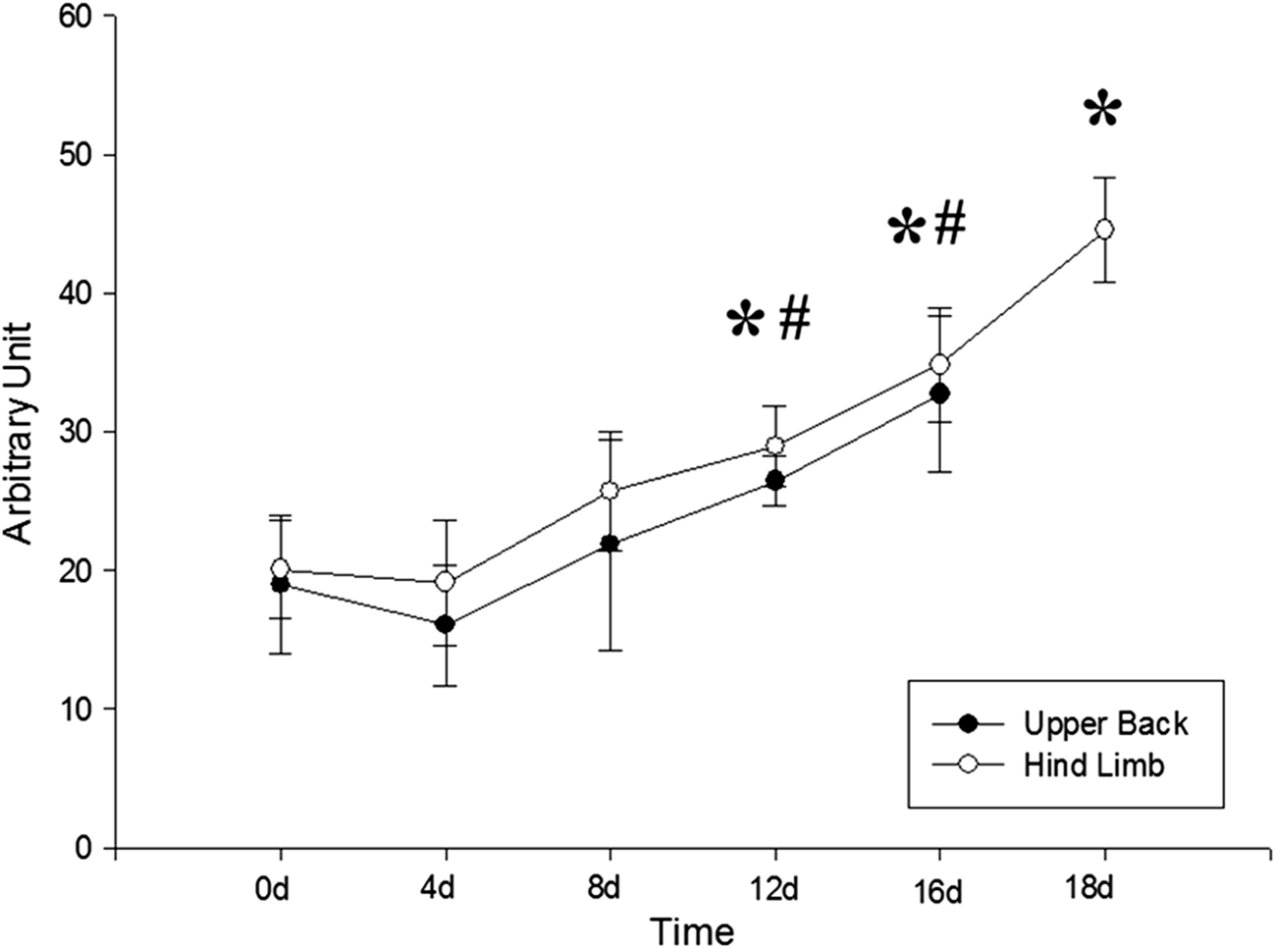
Video Intensity (VI) of silk gel implants increased over 18 days. The VI recorded at the hind limb group at day 12, 16 and 18 has significantly increased compared to that at day 0 after surgery. * *p *< *0.05* versus day 0 of hind limb group. At the same time, the VI recorded the upper back group at day 12, 16 has significance compared to day 0. ^#^*p *< *0.05* versus day 0 of upper back group

The silk gel implants appeared as a hypoechoic nodule with a well-defined boundary under the skin. The changes of the shape and echo intensity are clearly reflected by the 2D ultrasound imaging (Figs. 1, 2). The implants in the hind limb subcutaneous appeared as low-level gray and oval shape nodules in the first few days (Fig. 1a–d). The echogenicity of the silk hydrogel implants increased gradually showing by medium-level gray images, and the outline of nodules changed to thinner and flatter as time went by (Fig. 1e, f). The echogenicity of silk hydrogel implant was equal to the adjacent boundary and could not be clearly recognized until day 20 (Fig. 1g). The implants in rat’s back subcutaneous group degraded faster than those in the limb group (Fig. 2). In the rat’s back group, the implants were becoming thin and flat since day 4 (Fig. 2b–e), and could not be recognized at day 18 (Fig. 2f, g). Video intensity (VI) of silk gel implants measured by Image J software increased gradually in both groups (Fig. 3). Compared with the VI measured right after surgery, the VI acquired at day 12, 16 and 18 in both groups showed statistical significance (*p *< 0.05).

### Volume assessment of silk gel implants determined by three-dimensional US

**Fig. 4:**
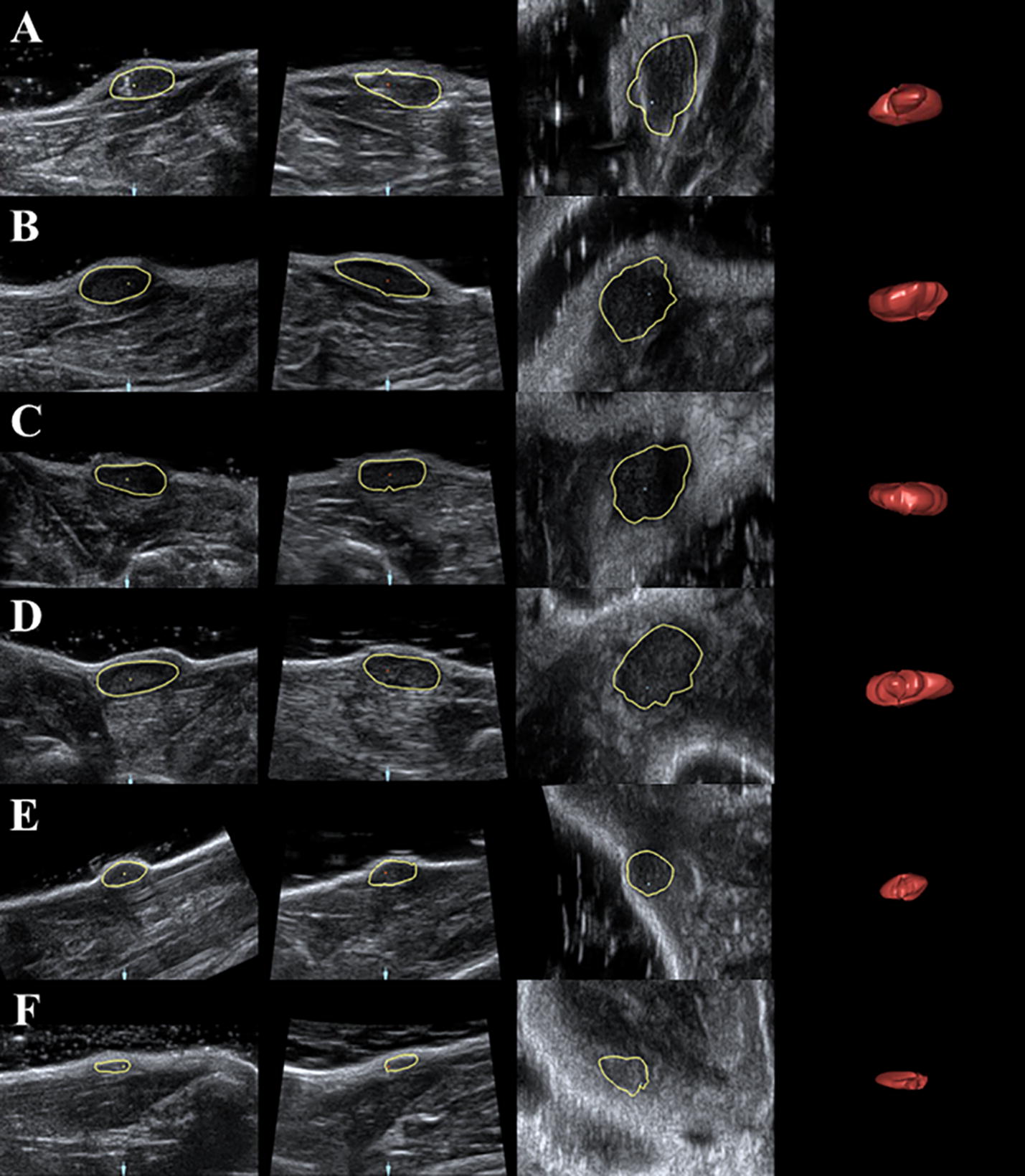
Silk fibroin implants at the hind limb subcutaneous at day 0, 4, 8, 12, 16 and 18 (**a**–**f**) observed by 4D View software

**Fig. 5:**
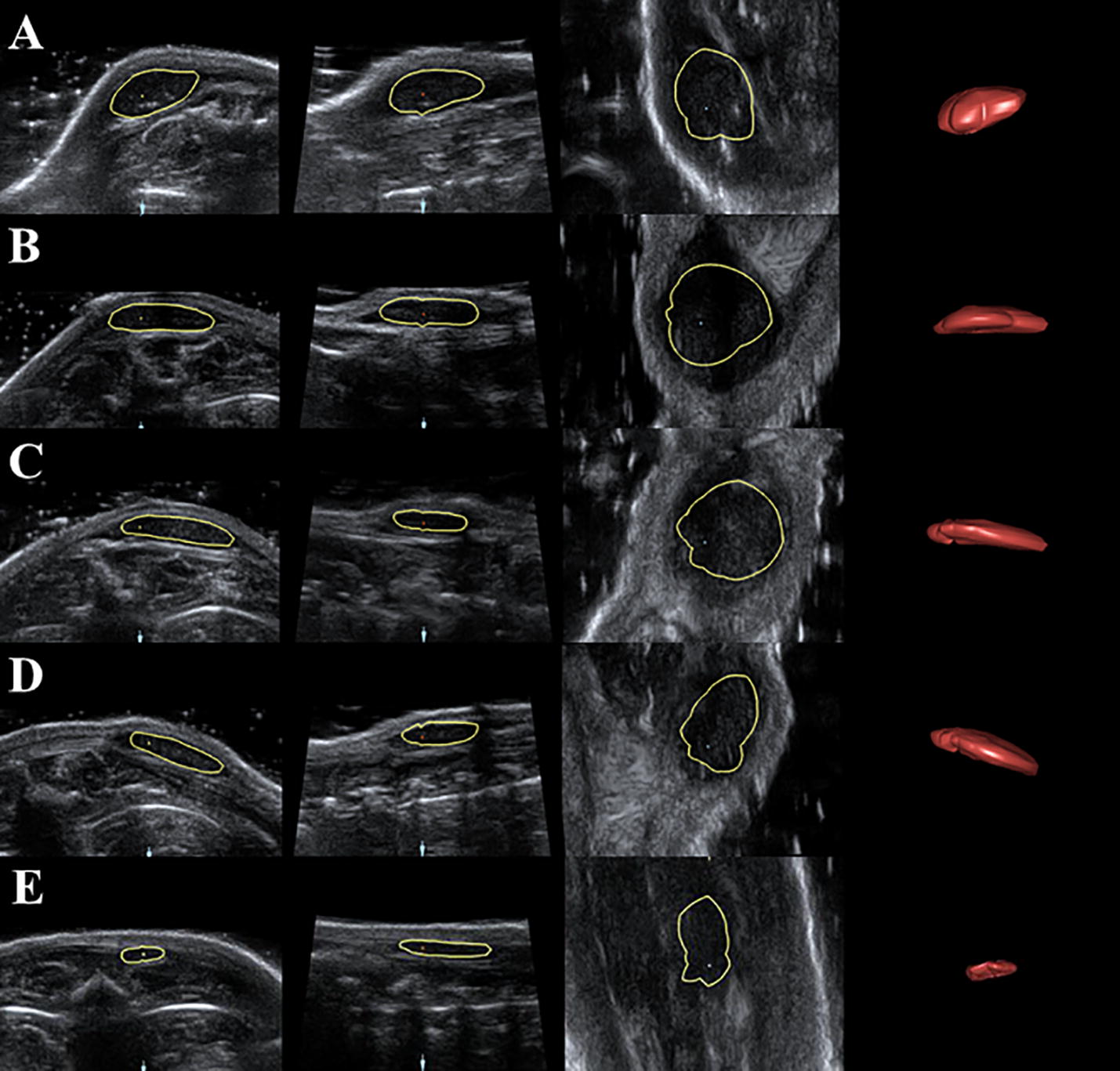
Silk fibroin implants at the upper back subcutaneous at day 0, 4, 8, 12, and 16 (**a**–**e**) observed by 4D View software

**Fig. 6:**
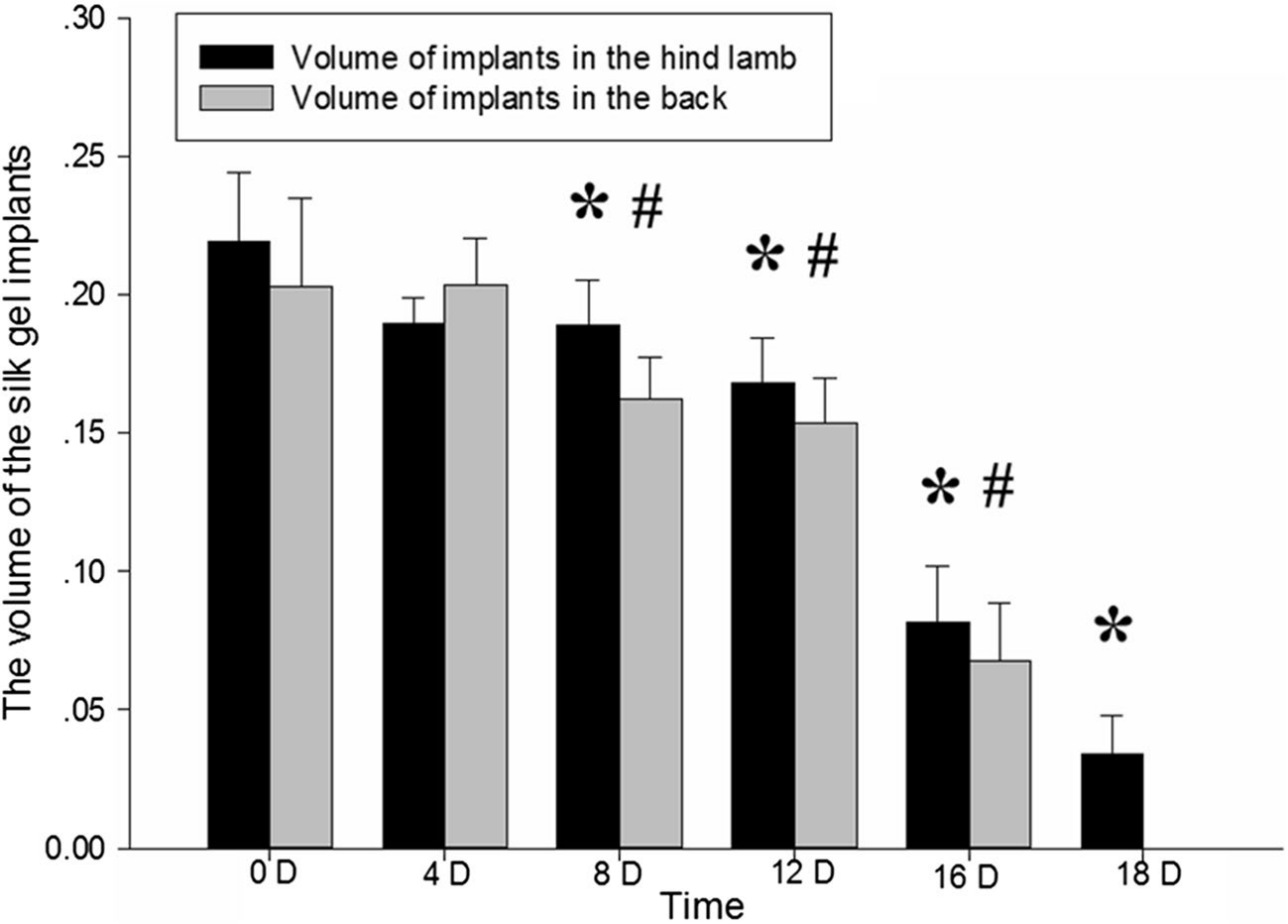
The volume of the implants was significantly reduced either in upper back group or hind limb group since day 8 compared with day 0 after implantation, *p < *0.05* versus day 0 of hind limb group. ^#^*p *< *0.05* versus day 0 of upper back group

The volumes of the gel implants at different time points were obtained and were calculated by 3D View software. The 3D images showed that the shape of the implants changed from hemispheroid into flat disk shape (Figs. 4, 5). After the contours of silk fibroin implants were delineated, the volumes were measured successfully at corresponding time point and had no significant change during the first 4 days after surgery, while reduced significantly either in upper back or hind limb group since the eighth day after surgery (Fig. 6). The volume of implants in rat’s back subcutaneous decreased faster than those in the limb group, which was consistent with the result of 2D US. Compared with the implants’ volume right after the surgery, significant changes (*p *< 0.05) were observed since the eighth day after implantation. The interobserver reliability for all three observers (ICC 0.996) indicated a good repeatability.

### Neovascularization determined by CEUS

**Fig. 7:**
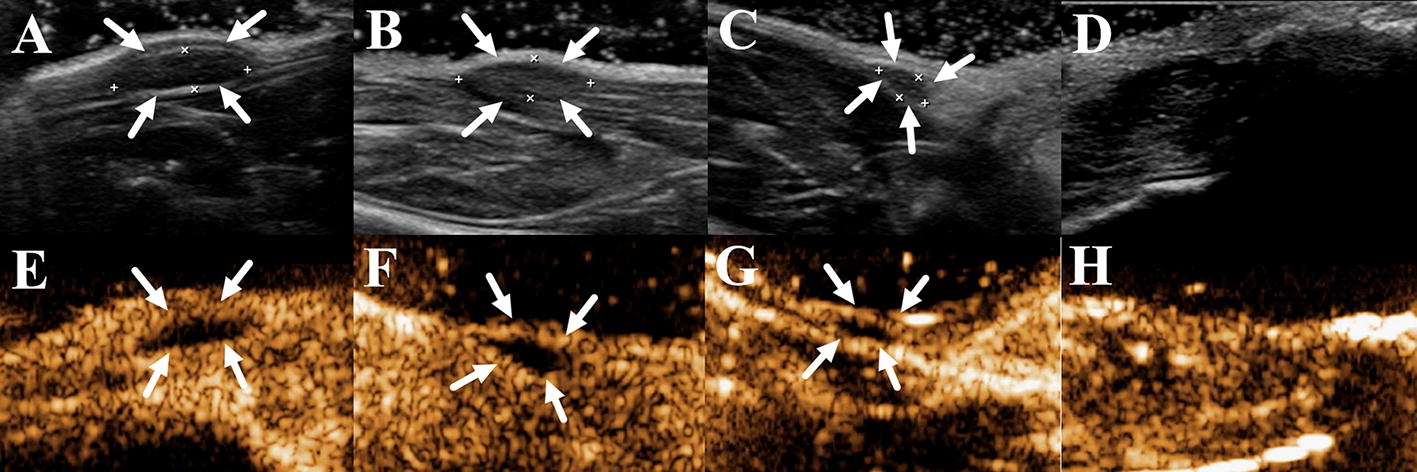
The silk gel implants observed by 2D (**a**–**d**) and CEUS (**e**–**h**) at day 12, 16, 18 and 20. As time went by, more microbubbles infused into the silk gel, indicating the progression of neovascularization. At day 20, microbubbles in the silk gels had a similar density to those in the surrounding tissues and the silk gel implants could not be distinguished (**h**), arrows, outline of silk hydrogel implants

**Fig. 8:**
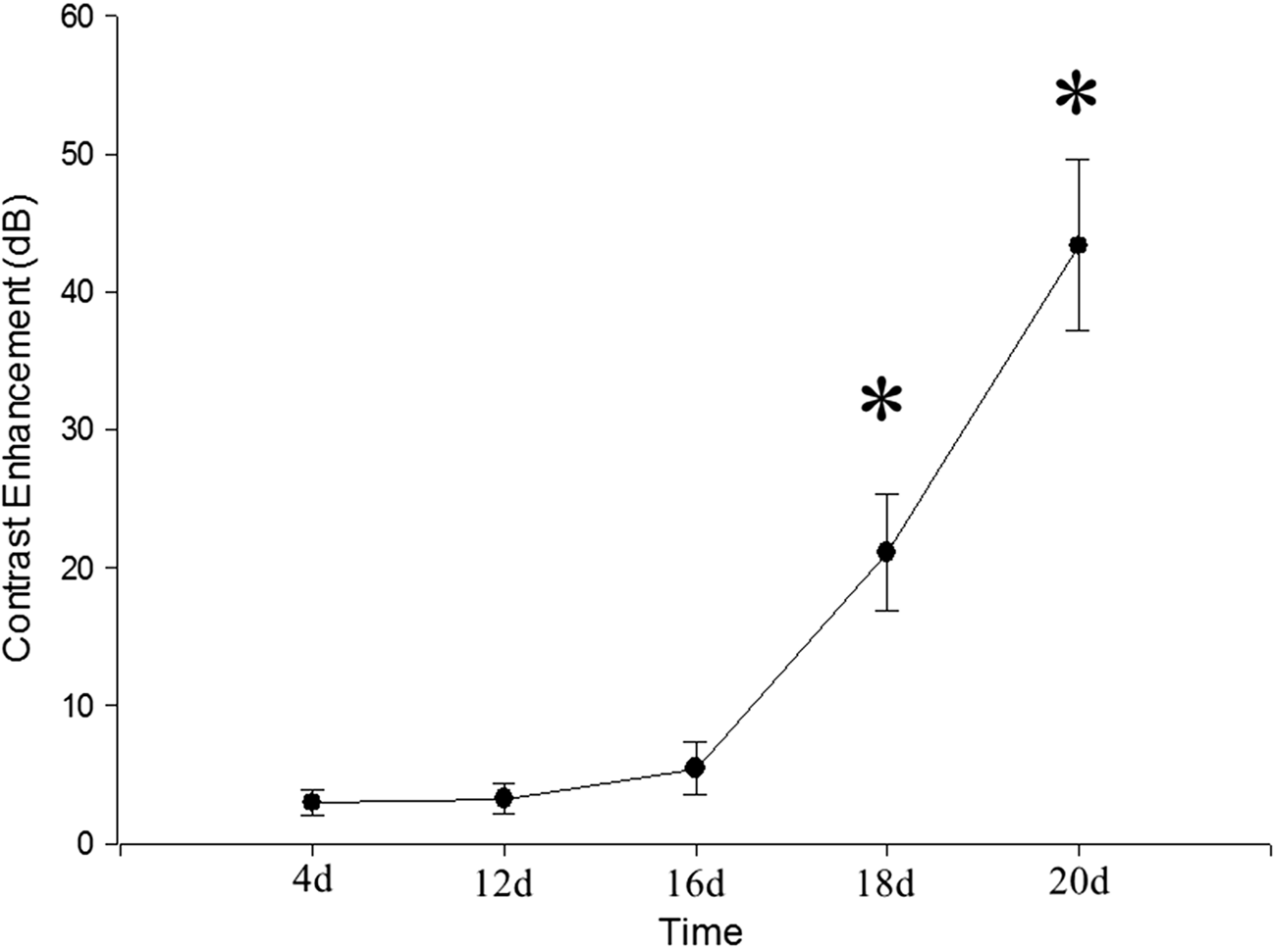
Contrast enhancement (dB) of silk hydrogel implants measured by CEUS. *Significant increases of dB for silk gel implants compared with day 4 (*p *< *0.05*)

In the contrast condition, the microbubbles opacified the vessels immediately after the injection and enhanced the tissues 6 s later. In the hind limb group, implant enhancement was not seen at day 4 after surgery and only a few microbubbles were shown at the edge of gel implant at day 12 (Fig. 7a, e). Microbubbles penetrated into inner regions of silk implants (Fig. 7b, f) since day 16. At day 20, all implants could not be identified by 2D US, and the acoustic characteristics of original implant sites were consistent with the adjacent tissues in the contrast mode (Fig. 7d, h). The regions of interest were delineated and the peak video intensity of the gel implants at each time point were obtained by the MCE software, which were displayed in Fig. 8. Compared to the contrast enhancement at day 4, there was a slight increase (< 5 dB) at day 12 and day 16, a moderate increase (20 dB) at day 18, and a significantly increase (< 40 dB) at day 20. The value at day 18 and 20 increased significantly compared with day 4 (*p *< 0.05).

### Histological analysis of silk gel implants

**Fig. 9:**
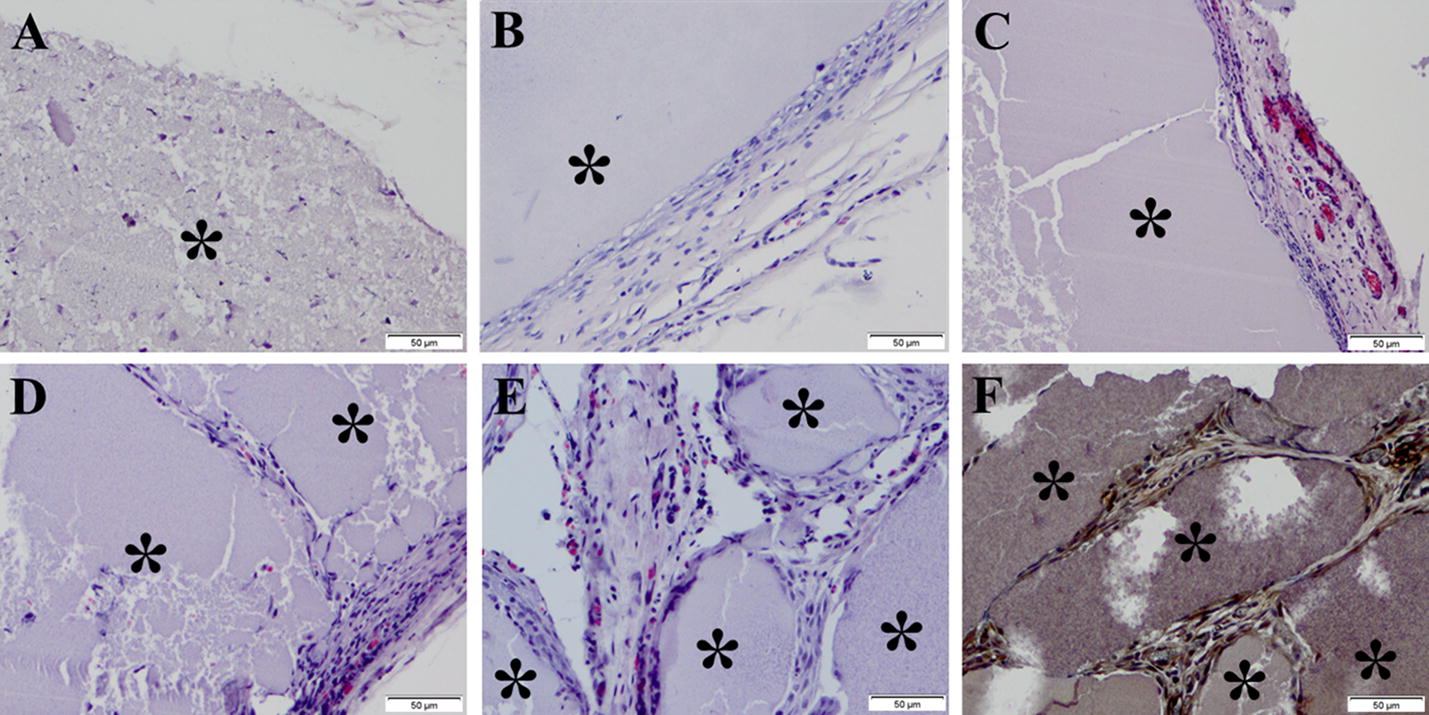
Histology of silk hydrogel implants. The gel implant samples at the hind limb subcutaneous at day 4, 12, 16, 18 and 20 (**a**–**e**) subjected to H&E staining and immunohistochemical staining at day 20 (**f**). At day 4, gel implants showed few cells attachment or tissue formation (**a**). A little of fibrous connective tissue had formed on the gel surface at day 12 (**b**), and micro vessels could be seen at day 16 and 18 (**c**, **d**). At day 20, a capsule membrane mainly consisting of blood vessels and fibrous connective tissue had formed on the gel surface or in the gel (**e**), and endothelial cell surface marker was expressed (**f**); *residual silk gel implants

After CEUS was performed, the gel implant samples were retrieved and analyzed histologically to corroborate the imaging data. The pathological results from upper back group and hind limb group underwent the same process, and the results from 5 representative time points are shown in Fig. 9. At day 4, gel implants showed a few cells infiltration (Fig. 9a). At day 12, a little fibrous connective tissue was shown on the gel surface (Fig. 9b) and a capsule membrane consisting of micro vessels was observed at day 16 (Fig. 9c), although the combination to the implant was not compacted. At day 18, the capsule membrane mainly consisting of blood vessels and fibrous connective tissue was becoming more compact around the gel (Fig. 9d). At day 20, large amount of blood vessels and connective tissues had formed and occupied all porous areas in the silk gel, although a few residual silk gel was still visible (Fig. 9e), and endothelial cell surface marker VIII was expressed by the immunohistochemical staining demonstrating the neovascularization (Fig. 9f).

### Transmission electron microscope analysis

**Fig. 10:**
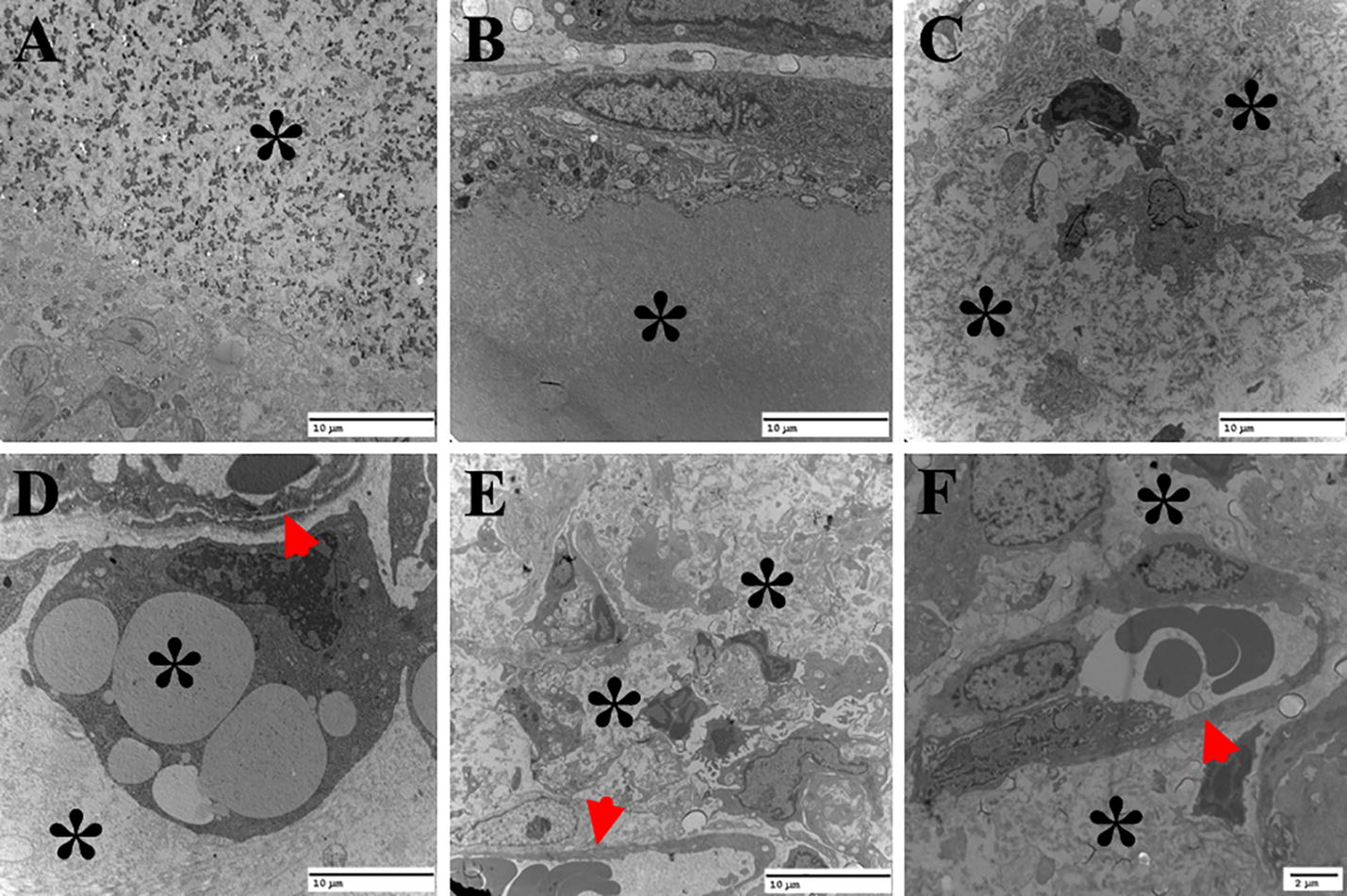
TEM images of silk gel implants observed at day 4 (**a**), 12 (**b**), 16 (**c**), 18 (**d**) and 20 (**e**, **f**). The inner cell density of the biomaterial increased over time (**a**–**c**), at day 18 and 20, only little silk fibroin gel was observed while blood vessel structure with endothelial and red blood cells was observed; *residual silk gel implants, arrows residual blood vessel

The TEM observation of silk implants is shown in Fig. 10. The silk gel implants had sparse density and only a few phagosomes were observed on the gels surface 4 days post surgery (Fig. 10a). At day 12, more and more phagosomes and phagocytes were observed at the border of the implants (Fig. 10b). At day 16, some granular leukocytes and fibroblasts were seen inside the implants (Fig. 10c). The amount of cells in the gel increased over time and some phagocytes with engulfed silk fibroin in the cell were observed at day 18 (Fig. 10d). At day 20, a large number of granular leukocytes, phagocytic cells and the vascular composed of epithelium and red blood cells were observed in the silk fibroin gels (Fig. 10e, f).

## Discussion

Biodegradable biomaterials play a key role in tissue regeneration and wound healing. Silk hydrogels are ideal materials for biomedical applications of tissue regeneration and drug delivery [21–25]. These mainly include sponge-like porous scaffolds for supporting proliferation and differentiation for bone regeneration [24], silk hydrogel for the repair of cancellous bone defects [26], and silk meshes and nanofibre mats as mechanical supports to repair damaged tissues in the body [27, 28]. As a result, there has been increasing interest in evaluating and controlling the rate of biodegradation of biomaterials quantitatively through in vivo animal trials [5, 29, 30]. Although silk hydrogels hold promise in regenerative medicine and for drug delivery, in vivo degradation of silk hydrogel has not been systematically investigated. The in vivo evaluation of implanted biomaterials mainly remains qualitative by descriptive observations for histological sections. All these studies were either qualitative or semi-quantitative. Recently, silk films were implanted indifferent tissues in rats and the in vivo degradation rates and tissue-regeneration rates were semi-quantitatively determined and correlated through histology, microscopy and image analysis methods [5, 31, 32].

As a non-invasive technique, ultrasound has high tissue resolution and repetitive applicability. Conventional 2D US has been used to characterize biomaterial properties changes in vivo. Heidmann et al. [6] applied a 7.5 MHz ultrasound transducer to detect the polylactide osteosynthesis fracture plate materials and monitor the degradation process over 36 months. In their study, ultrasound cannot display material structural changes and the growth of the surrounding tissues, but can be used to detect the degradation process by quantitative analyzing the change of the thickness of the material. However, the degradation of biomaterial is not even, which might cause the diameter change in 2D US, thus could not reflect the degradation rate precisely. In our study, ultrasound takes its advantage in the detection of the degradation of the silk hydrogels implants in vivo. The change of the echo intensity reflects the absorbing of the moisture in the implant and the progressing of proteolysis. In this study, in addition to 2D US, the 3D US was applied to monitor the contour and quantify the volume of silk hydrogels in vivo. The 3D volume obtained by the multi-planar image reconstruction established a complete 3D contour with 3D ultrasound volume automatic measurement technique. The decreasing of volume measured by 3D US proved the degradation and gradual absorption of the silk fibroin. The volume of the implants in either upper back or hind limb measured by 3D US was significantly reduced since day 8 compared with day 0 after implantation (Fig. 6). The video intensity of silk gel implants measured by 2D US increased since day 12 (Fig. 3). The 3D US could indicate the degradation of the silk implants earlier.

CEUS is a new technique that makes use of microbubble-based contrast agents to improve the echogenicity of the blood and thus improve the visualization and assessment of vessels and tissue vascularity [33, 34]. It can display vascular structures not only about the blood supply of the organization, but also be used to observe the vascularization of silk fibroin gel implants. The contrast enhancement recorded from implants of hind limb group was low in the first 16 days of implantation, due to the lack of angiogenesis in the gel. The contrast enhancement significantly increased at day 18 and the vascularization had progressed revealed by H&E staining and TEM (Figs. 9, 10). Thus, the accuracy of CEUS which was similar to TEM and histology in determining neovascularization can be provided for the entire hydrogel implant. Application of CEUS to detect silk fibroin gel implants can assess gel degradation process of neovascularization within the gel. The application of 2D US, 3D US and CEUS could detect the degradation and neovascularization process of the silk fibroin implants in vivo. The imaging techniques used in the present study can also be applied to other material formats that have defined shapes and relatively high US densities, such as porous scaffolds, solid plugs and films. Different implantation sites and animal species may also be explored in future studies.

Considering that the vascular density of soft tissue might contribute to the biodegradation rate, we embedded the silk gels in different spots in rat model. Although the biodegradation observed by 2D and 3D ultrasound of samples implanted at the back are faster than those of samples implanted at the hind limb. The histopathology confirmed the same process of neovascularization between the upper back group and hind lamb group. Therefore, the biodegradation process of silk hydrogel in the upper back subcutaneous was similar to that in the hind lamb subcutaneous, a faster biodegradation rate was obtained because upper back subcutaneous tissue has abundant vascular net [5].

Our study has its limitations. Due to the impacts of heart beating and respiratory movement, the contrast imaging was not steady and the result of the back implants contrast enhancement was excluded. Whereas the histology results revealed that the back implants experience the same neovascularization procedure with hind lamb group.

## Conclusion

Silk fibroin has good biocompatibility which can be used as ideal biomedical materials for surgical applications. The silk fibroin of 4% concentration under the skin degraded mainly in the way of hydrolysis and phagocytized in the first 16 days after implantation and degraded fast and formed the neovascular with microvessels during day 18–20. The biodegradation of silk hydrogels in the upper back subcutaneous has been shown to occur at a faster rate than in the hind lamb subcutaneous. Ultrasound is a promising technology to evaluate the degradation and neovascularization processes of silk hydrogels in vivo.
